# Anti-Inflammatory Activity of Antimicrobial Peptide Periplanetasin-5 Derived from the Cockroach *Periplaneta americana*

**DOI:** 10.4014/jmb.2004.04046

**Published:** 2020-07-05

**Authors:** In-Woo Kim, Joon Ha Lee, Minchul Seo, Hwa Jeong Lee, Minhee Baek, Mi-Ae Kim, Yong Pyo Shin, Sung Hyun Kim, Iksoo Kim, Jae Sam Hwang

**Affiliations:** 1 Department of Agricultural Biology, National Institute of Agricultural Sciences, Rural Development Administration, Wanju 55365, Republic of Korea; 2 College of Agriculture & Life Sciences, Chonnam National University, Gwangju 61186, Republic of Korea

**Keywords:** Periplanetasin-5, antimicrobial peptide, anti-inflammatory activity, cockroach, *Periplaneta americana*

## Abstract

Previously, we performed an *in silico* analysis of the *Periplaneta americana* transcriptome. Antimicrobial peptide candidates were selected using an *in silico* antimicrobial peptide prediction method. It was found that periplanetasin-5 had antimicrobial activity against yeast and gram- positive and gram-negative bacteria. In the present study, we demonstrated the anti-inflammatory activities of periplanetasin-5 in mouse macrophage Raw264.7 cells. No cytotoxicity was observed at 60 μg/ml periplanetasin-5, and treatment decreased nitric oxide production in Raw264.7 cells exposed to lipopolysaccharide (LPS). In addition, quantitative RT-PCR and enzyme-linked immunosorbent assay revealed that periplanetasin-5 reduced cytokine (tumor necrosis factor-α, interleukin-6) expression levels in the Raw264.7 cells. Periplanetasin-5 controlled inflammation by inhibiting phosphorylation of MAPKs, an inflammatory signaling element, and reducing the degradation of IκB. Through LAL assay, LPS toxicity was found to decrease in a periplanetasin-5 dose-dependent manner. Collectively, these data showed that periplanetasin-5 had anti- inflammatory activities, exemplified in LPS-exposed Raw264.7 cells. Thus, we have provided a potentially useful antibacterial peptide candidate with anti-inflammatory activities.

## Introduction

Insect antimicrobial peptides (AMPs) have been categorized into more than 150 types since the purification of cecropin in the hemolymph of pupae from *Hyalophora cecropia* in 1980. Insect AMPs are low molecular weight, cationic, and amphipathic compounds with variable length, sequence, and structure. They perform a pivotal role in the humoral immunity of insect innate immune systems against invading pathogens such as bacteria, fungi, parasites, and viruses. Most of the insect AMPs are induced rapidly in the fat bodies and other specific tissues of insects after septic injury or immune challenge. In such cases, the AMPs are subsequently released into the hemolymph to act against microorganisms [1-5]. Recently, various studies on AMPs have been conducted. For example, the Coprisin analog, CopA3, derived from dung beetles, has been shown to possess anticancer activity by causing apoptosis and necrosis in human gastric cancer cells [6, 7]. Scolopendrasin, an AMP isolated from *Scolopendra subspinipes mutilans*, plays a role in the treatment of anticancer and atopic dermatitis [8]. Therefore, invertebrates, including insects, are considered good sources for AMP selection.

The cockroach is one of the oldest winged insects and lives in contact with humans. Currently, there are about 4,000 species of cockroaches, of which 30 are known to be harmful to humans. Cockroaches generally exist in environments with pollutants, microbial toxins, insecticides, and other toxic substances, and they are considered surrogate hosts for microbes that cause many human diseases. Despite their generally destructive nature, cockroaches have recently been found to harbor potentially beneficial and medically useful substances. Notably, the adaptive methods of cockroaches to harmful environments, and the related physiologically active substances derived from their immune systems, are of value in various fields of research [9, 10]. In 2017, a powerful antimicrobial substance was found in the brain of cockroaches though identification and characterization [11]. Furthermore, according to studies by Hong *et al*., it was found that the periplanetasin-2 peptide derived fro cockroaches ameliorated pseudomembranous colitis caused by *Clostridium difficile* toxin A [12].

An inflammatory response occurs when immune cells detect exogenous physical or chemical stimulants or bacterial infection; it functions to restore or regenerate injured tissues by controlling various inflammatory mediators. Macrophages activated by excessive lipopolysaccharide (LPS) secrete nitric oxide (NO) and p*ro- inflammatory* cytokines, such as tumor necrosis factor-α (TNF-α) and interleukin-6 (IL-6). Furthermore, the expression of inflammatory substances, such as inducible nitric oxide synthase (iNOS) and cyclooxygenase-2 (COX-2), is increased. The genes and protein expression levels of *pro-inflammatory* cytokines are controlled by mitogen-activated protein kinases (MAPKs) and nuclear factor kappa B (NF-κB). MAPKs are known to mediate the transmission of inflammatory signals through the phosphorylation process. When NF-κB is activated in response to immune and inflammatory reactions, cytoplasmic NF-κB translocates to the nuclei of macrophages. The inhibited kappa B (IκB), originally bound to NF-κB, is degraded, and the NF-κB entering the nucleus plays an important role in the transcription of cytokines [13-16].

Recently, more research on the genome and transcriptome using next-generation sequencing is being conducted, alongside a growing interest in the various genetic characteristics and potential utilization of cockroaches. Previously, we performed an in silico analysis of the *Periplaneta americana* transcriptome. Among the AMPs identified, periplanetasin-5 was selected, and its antibacterial and hemolytic activities were characterized [9]. The current study aimed to investigate the anti-inflammatory activity of the periplanetasin-5 peptide in Raw264.7 macrophage cells.

## Materials and Methods

### Peptide

Periplanetasin-5 was synthesized using the solid-phase peptide synthesis method detailed by Anygen Co., Ltd. (Republic of Korea). The peptide was dissolved in acidified distilled water (0.01% acetic acid) and stored at -20°C until use.

### Cell Culture

Raw264.7 cells were maintained in the Dulbecco’s Modified Eagle Medium supplemented with 10% fetal bovine serum, penicillin G (100 U/ml), and streptomycin (100 μg/ml) (Invitrogen, USA). Cells were cultured at 37°C in a humidified incubator with 5% CO_2_.

### Cell Viability Assay

Raw264.7 cells plated in 96-well plates (2 × 10^4^ cells/well) were treated with periplanetasin-5 at varying concentrations (10, 20, 30, 40, 50, and 60 μg/ml). After a 24-h incubation period, cell viability was assessed using the Cell Titer 96 Aqueous One Solution Cell Proliferation Assay (Promega, USA) according to the manufacturer’s protocol. The optical density at 590 nm was measured with a microplate reader (Beckman DTX 8800 Multi- detector, USA).

### Nitric Oxide Assay

Raw264.7 cells were seeded at 2 × 10^4^ cells/well in a 96-well culture plate in assay medium and incubated for 24h. After incubation, cells were treated with different doses of periplanetasin-5 for 1 h. For the positive control, 0.1 μg of LPS solution was added to the wells, and for the negative control, an equal volume of distilled water was added. The plates were incubated for 24 h in a CO_2_ incubator before culture supernatant was collected from each of the wells. The production of NO in the culture media was measured using an NO Detection Kit (Intron, Republic of Korea). Nitrite levels were determined using a microplate reader at 550 nm (Beckman Coulter Co., USA). The percentage of NO production was calculated based on the LPS-treated sample as the maximum NO level.

### RNA Isolation and Quantitative Real-Time PCR (qRT-PCR)

Total RNA was extracted using TRIzol Reagent (Invitrogen) according to the manufacturer's instructions. One microgram of RNA was transcribed into cDNA using a High Capacity cDNA Reverse Transcription Kit (Applied Biosystems, USA). Quantitative real-time PCR (qRT-PCR) was performed using the specific primer set detailed in Table 1.

### Enzyme-Linked Immunosorbent Assay (ELISA)

Raw264.7 cells were seeded in 6-well plates (2 × 10^5^ cells/ml) and incubated for 24 h. After incubation, cells were treated with periplanetasin-5 in the absence or presence of LPS. After 24 h, the levels of IL-6 and TNF-α in 100 μl of culture media were measured using a Mouse TNF-α and IL-6 ELISA Kit (ThermoFisher, USA) according to the manufacturer's instructions.

### Western Blot Analysis

Cells were harvested and lysed in NP-40 lysis buffer (Fluka, USA) with protease and phosphatase inhibitors (ThermoFisher). Protein concentrations were measured using the Bradford Protein Assay Kit (Bio-Rad, USA). Equal amounts of protein were separated using 12% SDS-PAGE and transferred to PVDF membranes (Bio-Rad). Afterward, the membranes were blocked with 5% skim milk, incubated overnight at 4°C with each primary antibody, and then washed three times with TBST (25 mM Tris buffer, 0.15 M NaCl, 0.05% Tween 20). The membrane was incubated for 3 h at room temperature with the corresponding horseradish peroxidase (HRP)- conjugated secondary antibody and rewashed three times with TBST. Proteins were visualized using the ECL detection kit (Invitrogen). The antibodies used are as follows: Primary antibodies: rabbit anti-COX-2; rabbit anti- iNOS; rabbit anti-phospho and total-p44/p42 MAPK (Erk1/2); rabbit anti-phospho and total-p38 MAPK; rabbit anti-phospho and total JNK; rabbit anti-IkB (all antibodies mentioned above were from Cell Signaling Technology, USA). Secondary antibodies: mouse anti-β-actin (Sigma-Aldrich, USA); and HRP-conjugated secondary antibody (Promega).

### Neutralized LPS Measurement (Total Endotoxin)

Neutralized LPS measurements were obtained using the Pierce LAL Chromogenic Endotoxin Quantitation Kit (ThermoFisher) according to the manufacturer’s instructions. In brief, each concentration of periplanetasin-5 and LPS was incubated at 37°C for 30 min. Then, 50 μl of *Limulus* Amebocyte Lysate (LAL) was added and incubated for 10 min; the chromogenic substrate solution was added, and absorbance was measured at 405 nm.

### Statistical Analysis

Data are presented as mean ± standard deviation (SD) of at least three independent experiments. Differences among groups were evaluated by the Duncan post hoc ANOVA analysis and considered statistically significant at *p* < 0.05.

## Results and Discussion

### Cell Viability of Raw264.7 Cells Treated with Periplanetasin-5

**Fig. 1:**
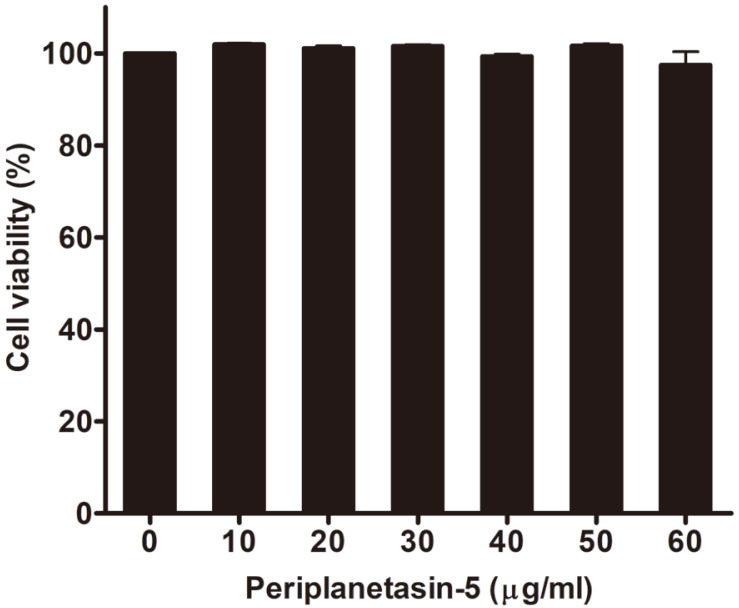
Cell viability of Raw264.7 cells after periplanetasin-5 treatment. Cells were treated with different concentrations (0, 10, 20, 30, 40, 50, and 60 μg/ml) of periplanetasin-5 for 24 h, and cell viability was measured by the [3-(4,5- dimethylthiazol-2-yl)-5-(3-carboxymethoxyphenyl)-2-(4-sulfophenyl)-2H-tetrazolium, inner salt; MTS].

To examine the cell viability of Raw264.7 cells treated with periplanetasin-5, cells were treated with various concentrations of periplanetasin-5 (10, 20, 30, 40, 50, and 60 μg/ml) for 24 h, and cell viability was measured through an MTS assay. As shown in Fig. 1, there was almost no change in cell viability. We found that periplanetasin-5 had no toxic effect on macrophages, and we therefore applied the same concentration range of periplanetasin-5 in the experiments that followed.

### Periplanetasin-5 Inhibits NO Production in LPS-Stimulated Raw264.7 Cells

**Fig. 2:**
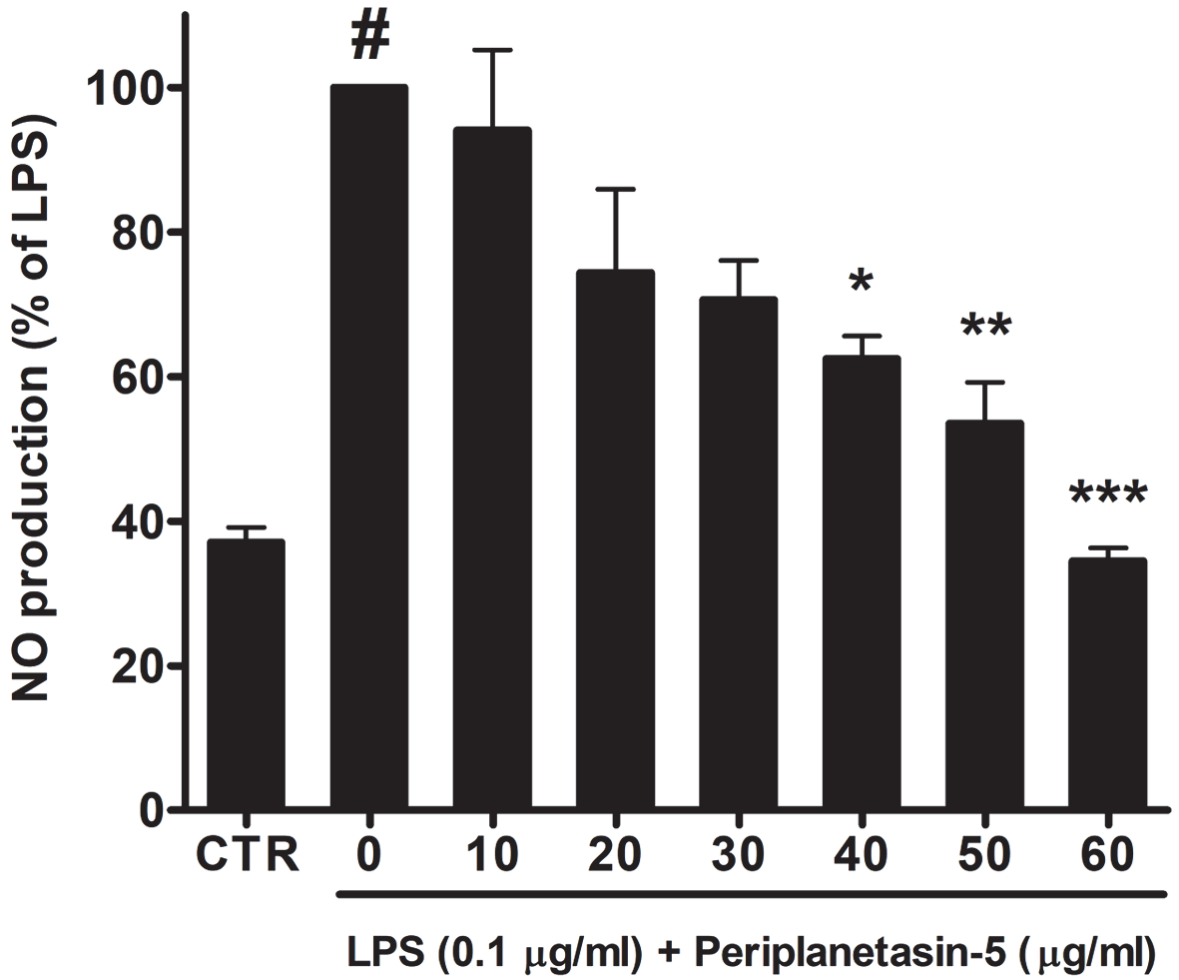
Nitric oxide (NO) production after periplanetasin-5 treatment. Cells were pretreated with periplanetasin-5 for 1 h prior to incubation with lipopolysaccharides (LPS) for 24 h. Data shown are means ± SD of triplicate experiments. #*p* < 0.05 compared to the control (CTR) group. ***p < 0.001, **p < 0.01, *p < 0.05 compared to the LPS group.

NO is an established signaling molecule involved in biological processes related to inflammation, and high levels are known to induce various inflammatory disorders. Thus, inhibition of NO production in the LPS- stimulated macrophage cell system is effective in controlling the inflammatory response [17]. In this study, periplanetasin-5 was found to reduce NO secretion in Raw264.7 cells stimulated with LPS in a dose-dependent manner (Fig. 2). Notably, compared to untreated samples, the amount of NO secreted by cells treated with periplanetasin-5 at 60 μg/ml was reduced by about 60%. The NO inhibitory effect of periplanetasin-5 is thought to be related to the expression of iNOS, a gene that affects NO production.

### Periplanetasin-5 Inhibits iNOS, COX-2 Expression and Production in LPS-Stimulated Raw264.7 Cells

**Fig. 3:**
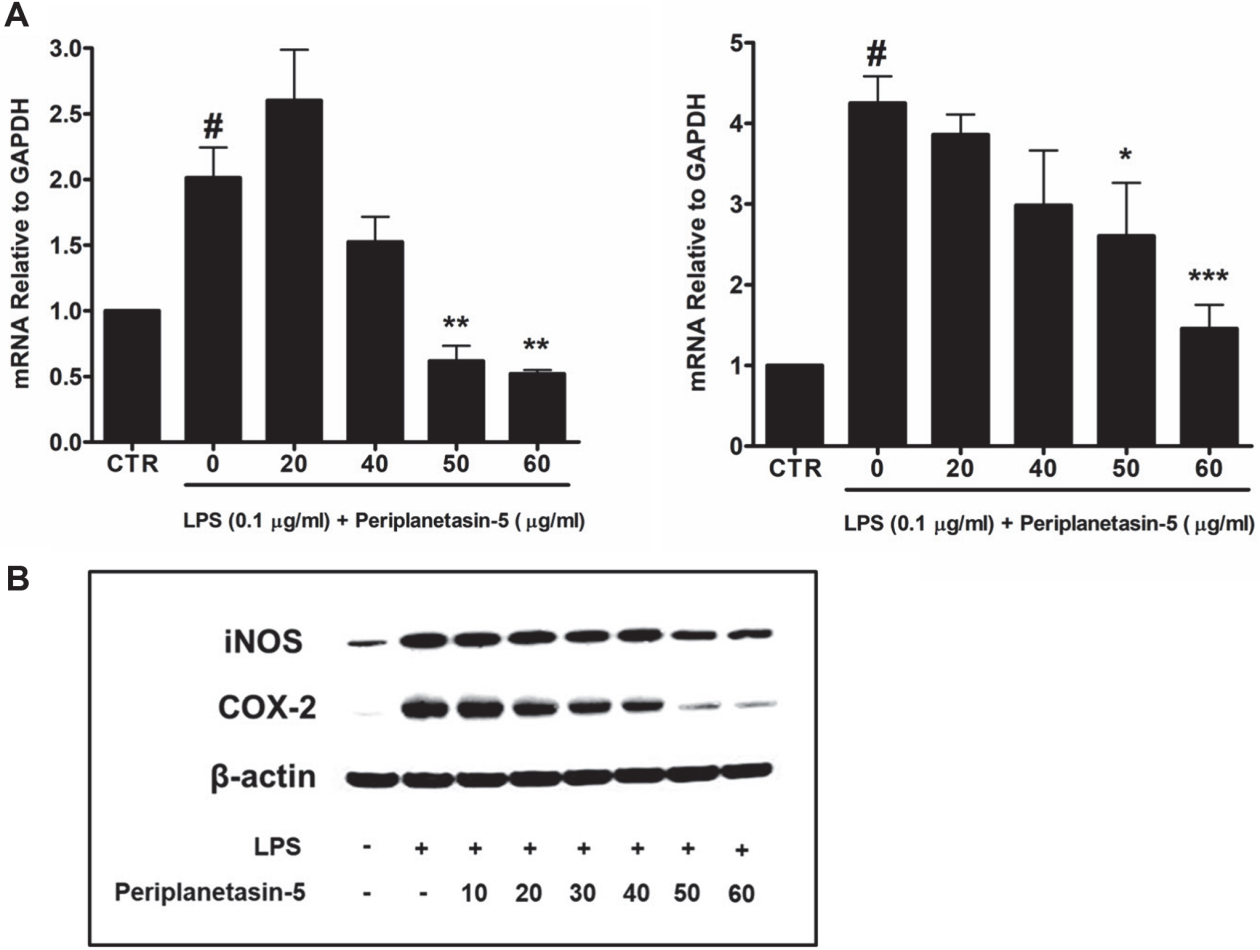
Inhibitory effects of periplanetasin-5 on iNOS and COX-2 expression in LPS-stimulated Raw264.7 cells. (**A**) The levels of iNOS (left) and COX-2 (right) mRNA were determined by quantitative real-time PCR. (**B**) The protein expression levels of iNOS and COX-2 were determined through western blot analysis. Data shown are means ± SD of triplicate experiments. #*p* < 0.05 compared to the control group. ***p < 0.001, **p < 0.01, *p < 0.05 compared to the LPS group. CTR, control; LPS, lipopolysaccharide.

We investigated periplanetasin-5 involvement in gene and protein expressions of iNOS and COX-2 through qRT-PCR and western blot analysis, respectively. As shown in Fig. 3A, treatment with LPS alone markedly increased iNOS and COX-2 gene expression, whereas treatment with periplanetasin-5 significantly inhibited iNOS and COX-2 gene expression. In addition, Fig. 3B shows that the relative protein expression was decreased in a dose-dependent manner after periplanetasin-5 treatment.

There are three types of NO synthase (NOS) that form NO: NOS-I (neural NOS, nNOS), NOS-II (inducible NOS, iNOS), and NOS-III (endothelial NOS, eNOS). Among these, iNOS does not normally exist in cells but is induced by stimuli such as LPS, cytokines, and bacterial toxins, or NF-κB activity, producing large amounts of NO over a long period. The produced NO acts as a carrier for inflammatory reactions and quickly reacts with superoxide in the body, thus activating macrophages as an inflammatory stimulator, along with other inflammatory substances. This leads to excessive inflammation, causing tissue damage [18-19]. Cyclooxygenases (COX) exist as two types, namely COX-1 and COX-2. While COX-1 exists in most tissues and is involved in normal physiology, COX-2 is rarely expressed under normal conditions. It is rapidly induced by stimuli such as cytokines and LPS and plays an important role in inflammatory responses [20-21]. Thus, the results showed that the decrease in gene and protein expressions of the inflammatory factors, iNOS and Cox-2, indicates that periplanetasin-5 has anti-inflammatory effects.

### Periplanetasin-5 Reduces the Expression of Pro-inflammatory Cytokines in LPS-Stimulated Raw264.7 Cells

**Fig. 4:**
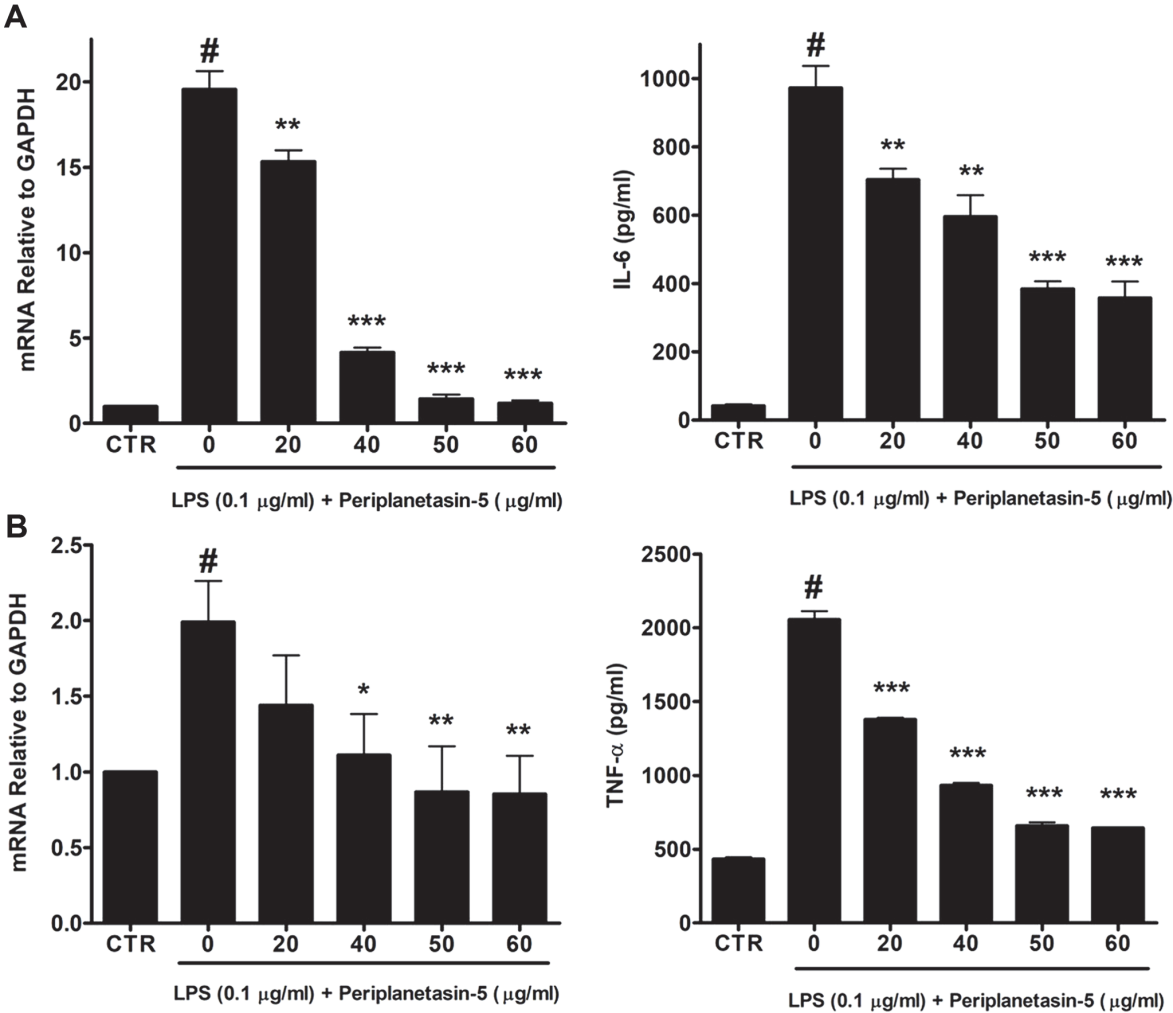
Inhibitory effects of periplanetasin-5 on the gene expression and production of pro-inflammatory cytokines in LPS-stimulated Raw264.7 cells. (**A**) The level of IL-6 mRNA was determined through quantitative real- time PCR (left) and the IL-6 production levels in the culture media were measured by ELISA (right). (**B**) The levels of TNF-α mRNA were determined through quantitative real-time PCR (left) and the TNF-α production levels in the culture media were measured by ELISA (right). Data shown are means ± SD of triplicate experiments. #*p* < 0.05 compared to the control group. ***p < 0.001, **p < 0.01, *p < 0.05 compared to the LPS group. CTR, control; LPS, lipopolysaccharide.

We evaluated the levels and gene expression of IL-6 and TNF-α produced in Raw264.7 cells to determine whether pro-inflammatory cytokines are reduced by periplanetasin-5 treatment. Treatment with periplanetasin- 5 at concentrations of 20, 40, 50, and 60 μg/ml significantly reduced the amount of IL-6 secreted in a dose- dependent manner (Fig. 4A). LPS-alone showed about 1,000 pg/ml IL-6, while periplanetasin-5 at 20 and 60 μg/ ml showed about 700 pg/ml and 380 pg/ml IL-6, respectively. Similarly, the gene expression of IL-6 tends to decrease as the periplanetasin-5 concentration increases. The gene expression of IL-6 was found to decrease rapidly when the periplanetasin-5 concentration was greater than 40 μg/ml. As shown in Fig. 4B, TNF-α secretion decreased in a dose-dependent manner when supplemented with periplanetasin-5 at 10 to 60 μg/ml. The total secretion of TNF-α was about 2,000 pg/ml during the LPS-alone treatment. However, it was 600 pg/ml when treated with 60 μg/ml of periplanetasin-5. The gene expression of TNF-α was shown to decrease to an untreated level with 60 μg/ml periplanetasin-5.

Inflammatory cytokines, such as IL-6 and TNF-α, are mediators of inflammatory reactions and are known to be involved in early inflammatory responses. The overproduction of these mediators is related to several inflammatory diseases and cancer [22]. TNF-α is an important inflammatory cytokine that engages in normal physiological immunity and inflammatory processes. However, TNF-α also plays a role in the development of chronic inflammation and related diseases following LPS stimulation. IL-6 is also considered a central inflammatory cytokine. Thus, controlling the production of these inflammatory intermediaries can be a very efficient tool for blocking the occurrence and progression of inflammatory diseases [22-24]. Therefore, periplanetasin-5 can effectively regulate the inflammatory response by controlling the secretion of IL-6 and TNF- α, which is likely to affect NF-κB and MAPK signaling similarly.

### Periplanetasin-5 Suppresses the LPS-Stimulated Phosphorylation of MAPKs in Raw264.7 Cells

**Fig. 5:**
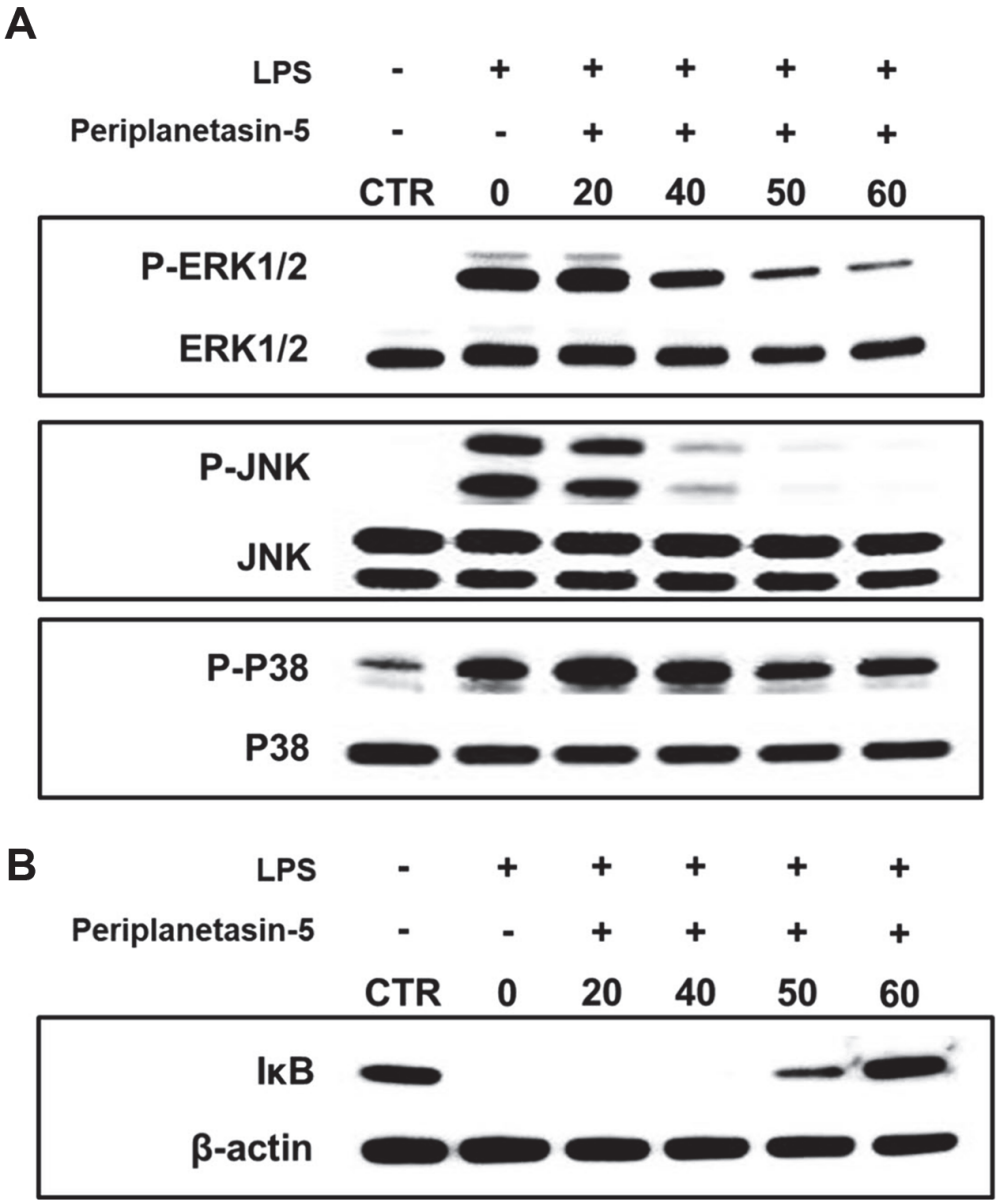
Inhibitory effect of periplanetasin-5 on MAPKs and NF-kB signaling pathways in Raw264.7 cells. (**A**) Raw264.7 cells were incubated with the indicated concentration of periplanetasin-5. Proteins were then isolated 30 min after LPS treatment, and phosphorylation of ERK, p38, and JNK was detected through western blot analysis. (**B**) Phosphorylation and degradation of IkB were detected through western blot analysis. CTR, control; LPS, lipopolysaccharide.

The phosphorylation of MAPKs and the control of NF-κB activity are important processes involved in chronic inflammation. We examined the effect of periplanetasin-5 on the activation of MAPK phosphorylation and IκB degradation using a western blot analysis. As shown in Fig. 5, the LPS-induced increases in p-p38, p-ERK, and p- JNK were significantly reduced by treatment with periplanetasin-5. The LPS-induced expression of p-p38 showed a significant decrease when treated with concentrations of 50–60 μg/ml. For p-ERK and p-JNK, there was a marked decrease in expression at concentrations of 40, 50, and 60 μg/ml. In the case of IκB, it was significantly degraded by LPS, but periplanetasin-5 efficiently inhibited LPS-induced IκB degradation. Notably, 60 μg/ml periplanetasin-5 recovered IκB to levels similar to those of the untreated group.

MAPKs (JNK; c-Jun N-terminal kinases, ERK; extracellular signal-regulated kinase, p38) are present in the cytoplasm in the unphosphorylated form; when stimulated with LPS, they are phosphorylated and translocate to the nucleus. The signal transmission path of MAPKs is known to play an important role in the activation of inflammatory reactions. Additionally, MAPKs are involved in the activation of transcription agents, including NF-κB and activator protein 1 (AP-1), which increase the secretion of inflammatory-related intermediates. NF- κB, an important signal transduction system molecule involved in immunity and inflammation, is a transcription factor that influences the expression of cytokines, growth factors, and cell adhesion molecules. When an external stimulus such as LPS is activated, the signaling pathway of the toll-like receptor 4 (TLR4) is activated, and IκB kinase (IKK) separates IκB and NF-κB, leading to the activation of NF-κB. At the same time, IκB is degraded. Activated NF-κB migrates from the cytoplasm to the nucleus, promoting the production of NO and various pro- inflammatory mediators, and thereby inducing a chronic inflammatory response [25-28]. Thus, these results suggest that periplanetasin-5 may have an anti-inflammatory effect through inhibition of the MAPK activation pathway and IκB degradation. For this reason, proper regulation of MAPK and IκB activity by periplanetasin-5 may be effectively applied to control various inflammatory diseases.

### Binding of Periplanetasin-5 to LPS in Solution

Recent studies have shown that a few AMPs have the potential to neutralize LPS-induced endotoxin effects, including LL-37, which was found in human white blood cells in 1995 [29]. LL-37 is a water-soluble peptide consisting of 37 amino acids. LL-37 was also found to play an important role in the defense against local and systemic infections by microorganisms while reducing inflammation. LL-37 also demonstrated direct binding to LPS and neutralizing biological activity because it inhibits IL-6, IL-1β, and LPS-induced TNF-α. According to Mu *et al*. [30], Cathelicidin-PP, originating from green frogs, is a membrane-targeting peptide with a rich base sequence showing strong antibacterial activity against bacteria and fungi. Moreover, it can partly neutralize LPS. These studies showed a direct correlation between the ability of AMPs to bind LPS and their anti-inflammatory activity [31-34].

**Fig. 6:**
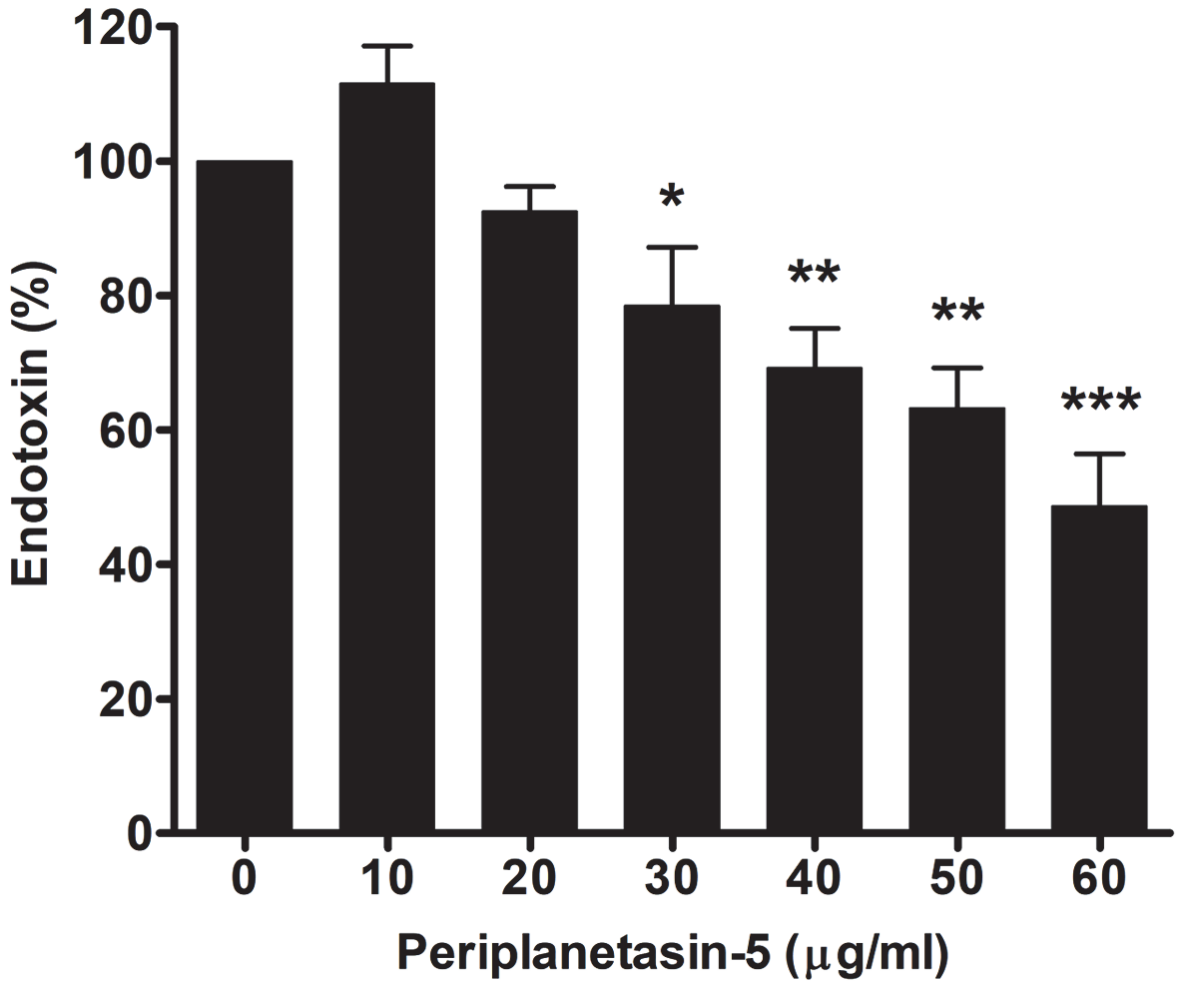
Periplanetasin-5 peptide neutralizes LPS in vitro. Various concentrations of periplanetasin-5 were incubated with LPS (0.4 ng/ml) for 30 min. After that, 50 μl of LAL was added and incubated for 10 min. Then, the chromogenic substrate solution was added, and absorbance was measured at 405 nm. A chromogenic LAL assay was used to evaluate the LPS (endotoxin) activity. Data shown are means ± SD of triplicate experiments. ***p < 0.001, **p < 0.01, *p < 0.05 compared to the LPS group. LPS, lipopolysaccharide.

Thus, we confirmed the binding ability of periplanetasin-5 and LPS with the LAL assay. The LAL testing method measures the endotoxins of the LPS. Depending on the amount of endotoxin, this method can indirectly measure the binding force between the LPS and AMP. As shown in Fig. 6, LPS endotoxin decreased in a dose- dependent manner in response to periplanetasin-5 treatment; at 60 μg/ml, the toxicity of LPS was inhibited by about 58%. Therefore, the inflammatory response induced by LPS in macrophages is estimated to limit the transmission of inflammatory signals by combining periplanetasin-5 with LPS.

LPS-induced inflammation in macrophages is initiated by the binding of LPS to TLR4 and is later known to result in the production of inflammatory cytokines, such as IL-6 via NF-κB [35]. Therefore, periplanetasin-5 binding of LPS can inhibit the coupling of LPS to TLR4, known as LPS decryption and neutralization. Peptides such as CP29 and Indolicidin are known to block LPS inflammatory signal transmission by competing with LPS for binding proteins, significantly reducing the release of TNF-α [33]. Similar to previously described AMPs, periplanetasin-5 isolates LPS and consequently prevents LPS-mediated TLR4 signals. Although the exact mechanism must be clarified, our results suggest that periplanetasin-5 directly interacts with LPS.

We demonstrated the anti-inflammatory activities of periplanetasin-5 in LPS-induced Raw264.7 macrophage cells. Respectively, periplanetasin-5 exerts significant anti-inflammatory functions by inhibiting the LPS-induced generation of NO, COX-2, and pro-inflammatory cytokines TNF-α and IL-6. The MAPKs (ERK, JNK, and p38) and NF-κB signaling pathways are involved in the anti-inflammatory effect. Furthermore, periplanetasin-5 caused partial neutralization of LPS in a dose-dependent manner. These results agree with those of previous studies that have identified the antimicrobial activity of periplanetasin-5. Therefore, periplanetasin-5 is an attractive candidate for both antimicrobial and anti-inflammatory treatments.

## Figures and Tables

**Table 1:** Primer sequences for real-time PCR.

cDNAs	Primer sequence	Accession No.
Cyclooxygenase-2 (COX-2)	Forward, 5'-CAGACAACATAAACTGCGCCTT-3'	NM_011198
	Reverse, 5'-GATACACCTCTCCACCAATGACC-3'	
Inducible nitric oxide synthase (iNOS)	Forward, 5'-CAGCACAGGAAATGTTTCAGC-3'	NM_010927
	Reverse, 5'-TAGCCAGCGTACCGGATGA-3'	
Iinterleukin-6(IL-6)	Forward, 5'-GAGGATACCACTCCCAACAGACC-3'	NM_031168
	Reverse, 5'-AAGTGCATCATCGTTGTTCATACA-3'	
Tumor necrosis factor-α (TNF-α)	Forward, 5'-ATGAGAAGTTCCCAAATGGC-3'	NM_013693
	Reverse, 5'-CTCCACTTGGTGGTTTGCTA-3'	
Glyceraldehyde-3-Phosphate Dehydrogenase (GAPDH)	Forward, 5'-AAGGTCATCCCAGAGCTGAA-3'	NM_008084
	Reverse, 5'-CTGCTTCACCACCTTCTTGA-3'	
